# Balancing the safeguarding of privacy and data sharing: perceptions of genomic professionals on patient genomic data ownership in Australia

**DOI:** 10.1038/s41431-022-01273-w

**Published:** 2023-01-11

**Authors:** Yuwan Malakar, Justine Lacey, Natalie A. Twine, Rod McCrea, Denis C. Bauer

**Affiliations:** 1 Responsible Innovation Future Science Platform, Commonwealth Scientific and Industrial Research Organisation (CSIRO), Brisbane, Queensland Australia; 2 Transformational Bioinformatics, Commonwealth Scientific and Industrial Research Organisation (CSIRO), Sydney, Australia; 3 Applied BioSciences, Faculty of Science and Engineering, Macquarie University, Macquarie Park, Australia; 4 Department of Biomedical Sciences, Faculty of Medicine and Health Science, Macquarie University, Macquarie Park, Australia

**Keywords:** Ethics, Health policy

## Abstract

There are inherent complexities and tensions in achieving a responsible balance between safeguarding patients’ privacy and sharing genomic data for advancing health and medical science. A growing body of literature suggests establishing patient genomic data ownership, enabled by blockchain technology, as one approach for managing these priorities. We conducted an online survey, applying a mixed methods approach to collect quantitative (using scale questions) and qualitative data (using open-ended questions). We explored the views of 117 genomic professionals (clinical geneticists, genetic counsellors, bioinformaticians, and researchers) towards patient data ownership in Australia. Data analysis revealed most professionals agreed that patients have rights to data ownership. However, there is a need for a clearer understanding of the nature and implications of data ownership in this context as genomic data often is subject to collective ownership (e.g., with family members and laboratories). This research finds that while the majority of genomic professionals acknowledge the desire for patient data ownership, bioinformaticians and researchers expressed more favourable views than clinical geneticists and genetic counsellors, suggesting that their views on this issue may be shaped by how closely they interact with patients as part of their professional duties. This research also confirms that stronger health system infrastructure is a prerequisite for enabling patient data ownership, which needs to be underpinned by appropriate digital infrastructure (e.g., central vs. decentralised data storage), patient identity ownership (e.g., limited vs. self-sovereign identity), and policy at both federal and state levels.

## Introduction

“*Genomics has the potential to reshape clinical practice and to fundamentally change the way we prevent, diagnose, treat and monitor illness, providing the opportunity to have more precise and tailored treatments*.” ([1], p. 2)

The above passage from the Australian National Health Genomics Policy Framework (National Framework hereafter) summarises a range of benefits that clinical genomics can offer to healthcare. The National Framework recognises the role that responsible collection, storage, use, and management of genomic data plays in realising these benefits. Establishing responsible data governance is one of the five strategic priorities identified in the National Framework. Two action items of this strategic priority are of high relevance to this paper. The first action envisions safeguarding the “privacy” of individuals whose genomic data are collected and stored. The second action supports the promotion of “data sharing” for research with national and international genomic alliances to advance health and medical science. There are, however, inherent complexities to achieving these two priorities, both within Australia and in other health jurisdictions.

The World Economic Forum [2] identifies an ethical tension implicit between managing patient privacy and sharing genomic data for research. Data privacy requires keeping individuals’ genomic information securely protected, and an inability to do this could have irreparable consequences for patients [3, 4]. Absolute privacy, however, limits data sharing for biomedical research, which consequently slows progress in understanding the relationship between human genes and our health and well-being [5, 6]. Additionally, data sharing has been identified as key to advancing precision health and tackling health inequities by increasing diversity in global genome databases [7, 8]. This ethical tension is not unique to Australia and similar issues are prevalent in all continents that seek to collect and share genome data for medical advancement. Such an example is the European “1+ Million Genomes” Initiative, which aims to collect and enable federated genome data access across the European Union.

The risk to patient privacy is one of the most widely examined ethical subjects in the field of clinical genomics [9, 10]. Genomic data can be identifiable [11], and patients are concerned about potential misuse [12] that could lead to potential discrimination [13] by insurance providers or prospective employers, for example. Although there are strong recommendations around safeguarding patients’ privacy, data breaches can occur by accident as a result of human error or poor security protocols [14].

Establishing patient data ownership, enabled by blockchain technology, is increasingly suggested as the solution to managing data sharing and the safeguarding of patients’ privacy [15, 16]. Such platforms enable “self-sovereign identity”, which allow patients to own, control, and decide by who, how and for what purpose their data will be used [16, 17]. Although patient data ownership sounds promising, implementing it requires a number of considerations. For example, Ballantyne [18] argues the term “ownership” implies property ownership, which is problematic within publicly funded health systems. She argues that in national health systems, like Australia, genomic data are co-produced by hospitals and laboratories, and the resources are funded by taxpayers, meaning there can be no single point of ownership. Furthermore, ownership also means the owner has rights to sharing data (as property) and benefiting from it [19, 20], which, again, adds another level of complexity in the current modality of the health system.

In Australia, patients’ genomic data are considered sensitive medical information and are protected with the highest priority under the Commonwealth Privacy Act 1988. Individual states are responsible for managing patients’ health data in Australia, and there is a wider call for nationally federated data governance [21, 22]. Efforts are underway to build a national approach to managing genomic data [23].

The need for patients to have a greater control over their data has been well-argued in the literature [24, 25] and in global forums such as the Global Alliance for Genomics and Health [26]. The Australian Genomics Health Alliance has piloted a platform to trial a dynamic consent approach, in which patients are given authority to make decisions about their participation and use of their data in research studies [27]. Similar efforts have been trialled in European countries, for example, Cooperative Health Research in South Tyrol [28] and Dwarna in Malta [29]. However, centralised platforms can pose security risks arising from the handling of large volumes of data, and they are less able to facilitate active patient participation [30, 31]. By contrast, self-sovereign identity is user-centric and need not rely on a centralised system [32, 33]. This means the data can be owned by the patient in a decentralised system, and any request to access the data should be approved by the patient [34].

Clinical genomics in Australia is identified as a “complex adaptive system” [35], in which multiple actors and institutions play specific roles [36–38]. As the concept and practice of patient data ownership is still emerging, we undertook this study to explore the views of professionals who play key roles in facilitating clinical genomics. It is not our intention to promote any particular model of patient data ownership but to respond to the growing literature on this topic by presenting the professional perspectives and experiences of those working within the clinical genomic system as a legitimate and valuable contribution to the broader discussion within this complex system.

Given the growing literature on the use of blockchain technology for managing patient data ownership, this research aims to provide insights into how professionals working in Australian clinical genomics view the barriers and opportunities of patient data ownership.

## Materials and methods

### Research design

The study was designed to engage with four groups of genomic professionals, namely clinical geneticists, genetic counsellors, bioinformaticians, and researchers (their roles are summarised in the Supplementary Information, Section 1). Having input from these four groups of professionals provides a system-level view of the professional delivery of a range of services across the clinical genomics system. Engaging with a diverse set of professionals in clinical genomics also allowed us to examine any similarities and differences in their views, and how their professional roles and responsibilities might inform their views on patient data ownership.

We applied a mixed methods approach to design the data collection and analysis; an online survey was conducted to collect both qualitative and quantitative data simultaneously. The qualitative data served the purpose of “complementarity”; that, is to elaborate or clarify the results from quantitative data [39]. A set of questionnaires was developed, pretested, and commissioned through an online platform, QuestionPro. Informed consents were obtained from all respondents. This research was approved by CSIRO’s Social Science and Interdisciplinary Human Research Ethics Committee (093_20).

### Survey design and data collection

The survey included questions about potential benefits, risks, and governance of clinical genomics in Australia. Only questions relating to patient data ownership were included, in accord with the scope of this paper. We asked to what extent respondents agreed that patients deserved rights to: (1) own their genomic data; (2) control the use of their genomic data; and (3) decide who uses their genomic data. We divided patient data ownership into these three items to explore whether respondents differentiated data ownership from patients controlling and making decisions about their data. As our intention was not to prescribe a particular model of patient data ownership, no detailed description of these three levels of patient data ownership was provided to respondents. This also allowed us to explore various assumptions and beliefs that participants brought to these concepts.

The questions were measured on a scale from 1 to 8, where 1 = Strongly disagree, 4 = Neither agree or disagree, 7 = Strongly agree, and 8 = Do not know. These scale questions were followed by an open-ended question, asking the reason for any disagreement, which was triggered when a participant expressed disagreement with any of the three data ownership items above. The reason for exploring disagreement in detail is because these motivations are currently underexamined in the literature. We also asked respondents to identify a realistic timeframe by which they believed patient data ownership might be implemented in Australia. Following this, another open-ended question asked respondents to identify any critical barriers and enablers to implementing patient data ownership.

As we were targeting a specific set of professionals, we sought support from professional societies, such as Australian Genomics, Human Genetics Society of Australasia, Australasian Genomic Technologies Association, Australian Bioinformatics and Computational Biology Society, and state-based genetic clinics to recruit respondents. Further, we used a snowball approach [40], requesting respondents to share the survey in their networks. To maintain the privacy and confidentiality of respondents, the survey was anonymous.

### Data analysis

All data analysis was performed in R software [41]. For quantitative data, we ran a descriptive analysis (frequency, mean, and standard deviation) to explore how different groups of professionals perceived patient data ownership across the three items. This suggested differences in perceptions between the types of professionals, so we combined the three ordinal-level items into an interval-level average measure to test the hypothesis that the perceptions of these professionals were not statistically significantly different from each other. An ANOVA test followed by Tukey’s *t*-tests were performed at the 95% confidence level, and the packages used for quantitative data analysis and data visualisations are provided in the Supplementary Information (Supplementary Table 1).

Thematic analysis was performed on the qualitative data, using a coding framework to ensure systematic identification of themes [42]. Textual data from respondents who disagreed patients should either own or control or decide who uses their data were analysed. Similarly, textual data relating to the enablers and barriers in implementing patient data ownership were analysed. In both analysis, codes were, first, identified and then, themes were generated from similar meaning codes. For coding reliability, two researchers were involved in thematic analysis [43].

## Results

### Australia’s genomics professionals were generally favourable towards patient data ownership

A total of 184 respondents completed the survey, though only 117 respondents across the four groups of professionals completed the questions relevant for this analysis. Among them were 50 genetic counsellors, 30 researchers, 20 bioinformaticians, and 17 clinical geneticists. The respondents were distributed across six states and two territories in Australia. Most respondents were from New South Wales (43), followed by Victoria (29), Queensland (20), Western Australia (11), South Australia (9), Australian Capital Territory (3), Northern Territory (1), and Tasmania (1).

**Fig. 1:**
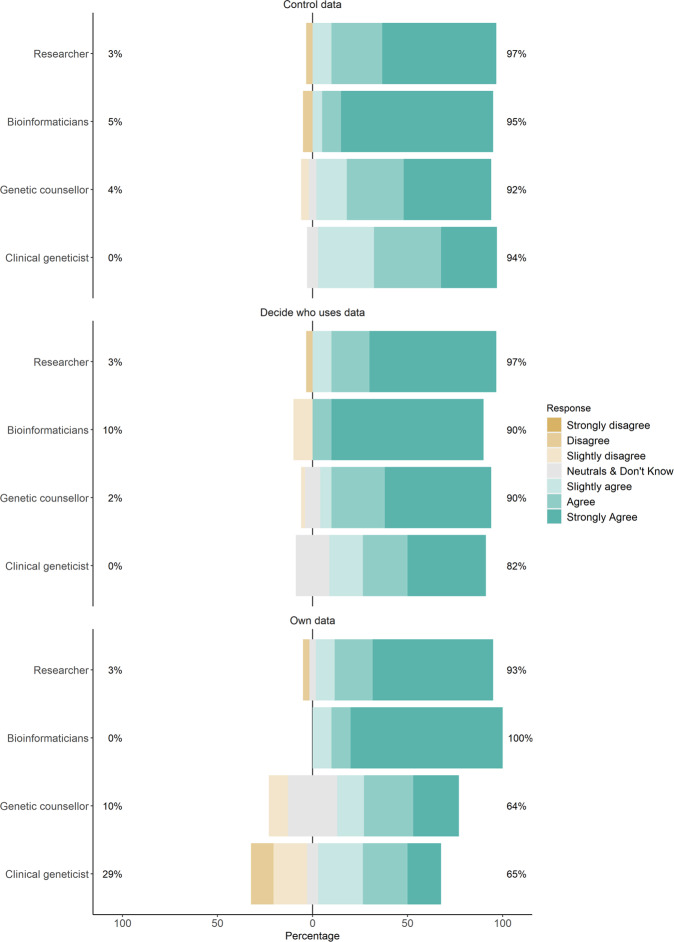
Perceptions towards patient data ownership by professional groups. Percentages on the right and left represent the percentages of respondents agreeing and disagreeing to the three items of patient data ownership respectively.

Respondents rated their perceptions of patient data ownership on a scale from 1 to 8. We merged “neither agree or disagree” and “don’t know” to create a new scale item of 4 = “neutrals & don’t know” to maximise the sample size for the analysis and for calculating means. We investigated whether the perceptions on each item of patient data ownership varied among the four professional groups. The results showed most respondents (>50%) across all professional groups had a favourable perception towards patient data ownership (Fig. 1). Over 90% of bioinformaticians and researchers agreed on all the three items. However, the responses of clinical geneticists and genetic counsellors were more varied across the three items, especially on patients’ rights to own data. Nearly one-third of clinical geneticists responded that patients should not have rights to own their data, and more than a quarter of genetic counsellors (26%) remained neutral on this question. These results indicated that respondents’ professional roles, experience, and responsibilities may influence how they view the benefits and drawbacks of patient data ownership.

### Support for patient data ownership decreases among professionals with higher degrees of direct patient interaction

**Fig. 2:**
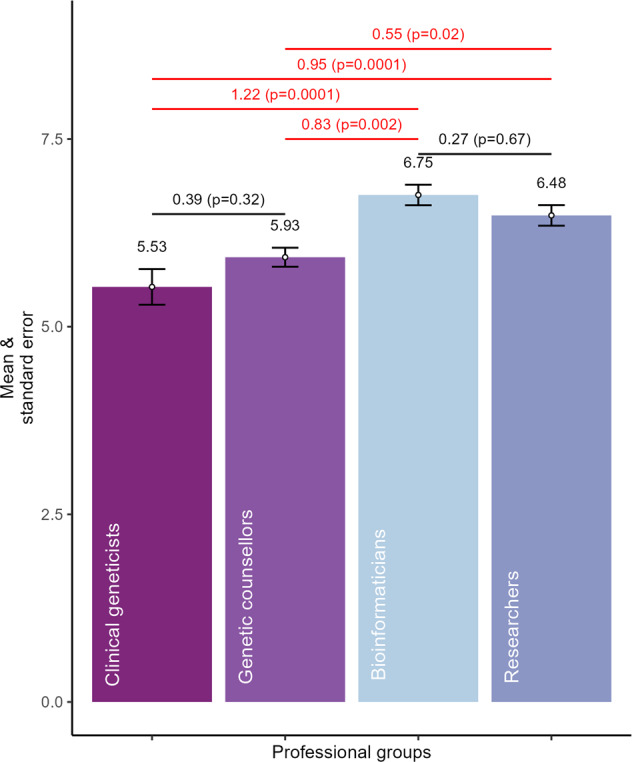
Test of significance between four professional groups. The bars show the means and their standard errors for perceived patient data ownership. The results of Tukey’s post hoc tests are shown above the mean values, with the differences in means and the *p* values in the parentheses. For example, the difference in means between clinical geneticists and genetic counsellors is 0.39 and a *p* value of 0.32. The red horizontal lines show statistically significant results, while the black lines were not significant.

The means of perceptions towards patient data ownership across the three scale items were computed. The mean of the three items of patient data ownership created an internally inconsistent single measure of perceptions towards patient data ownership (alpha = 0.79). Then we identified any outliers present in the data that may influence the analysis [44]. After removing three outliers (one researcher, one bioinformatician, and one genetic counsellor) to meet normal distribution assumptions for hypothesis testing, a total of 114 observations were used for this analysis. The mean values across the four professionals are shown in Fig. 2 (Also in Supplementary Table 2). Since 4 was the scale midpoint (neutrals & don’t know), any mean values over 4 represented agreement and values below 4 disagreements, on average. All professional groups were supportive of patient data ownership, as the means were above 4. The mean values were the lowest among clinical geneticists followed by genetic counsellors and highest among bioinformaticians and researchers. This finding further indicated a potential influence of professional roles, experience, and responsibilities on their perceptions.

To test whether the perceptions of professional groups towards patient data ownership varied according to their professional roles and responsibilities, we performed a hypothesis testing:

- Null hypothesis (H_0_) = the mean perceptions of all professional groups are same.
- Alternative hypothesis (H_A_) = the mean perceptions of all professional groups are different.

The results of the ANOVA test confirmed that the perceptions of patient data ownership were statistically different among the professional groups at the 95% confidence level (*p* = 0.000014) (Supplementary Table 3); hence, H_A_ was accepted. Further Tukey’s significance tests were used for post hoc comparison of means. The difference between means and their *p* values (significance level) are shown in Fig. 2; the Tukey test results are provided in the Supplementary Information (Supplementary Table 4). The results show that the difference in perceptions towards patient data ownership between clinical geneticists and genetic counsellors was not statistically significant (*p* = 0.32). A similar result was found between bioinformaticians and researchers (*p* = 0.67). Whereas statistically significant differences in perceptions towards patient data ownership were found between (1) clinical geneticists and bioinformaticians (*p* = 0.0001) (2), clinical geneticists and researchers (*p* = 0.0001) (3), genetic counsellors and bioinformaticians (*p* = 0.002), and (4) genetic counsellors and researchers (*p* = 0.02). This finding suggests that professionals with no direct interface with patients (i.e., working “behind-the-scenes”) have more favourable perceptions towards patient data ownership than “patient-facing” professionals.

### Thematic analysis

#### Exploring the nature of disagreement with patient data ownership

Fifteen respondents expressed disagreements towards patients having rights to either own or control or decide who uses their data, of which, 14 respondents provided written comments about the reasons for their disagreement. We identified one major theme that explains the reasons for their disagreement, which was “control but not own”.

The theme of “control but not own” captured the views that patients have rights to control who uses their data, but this does not automatically translate to the ownership of that data. This was expressed by one genetic counsellor as follows: “I don’t think they [patients] need to own the data, just own the right to make decisions on what is done with it” (G14). These respondents also identified three challenges they saw being related to patients “owning” their data: “copyright”; “collective ownership”; and “potential misuse”.

According to the Copyright Act 1968 of Australia, the copyright of genomic data rests with the compiler that has invested resources (human and financial) in collecting, validating, and storing the data. In a similar vein, one genetic counsellor stressed, “data is generated by the lab and therefore is theirs to own/manage, with responsible, pre-agreed terms that patients consent to at [the] time of test” (G1).

Respondents also reflected the perception that a patient’s genomic data is subject to the collective ownership of the patient’s family. One researcher described why it would be wrong for one person to hold ownership of their genomic data, “I believe my genome is not mine but my parents’ and their parents’ so looking at my genome as my own is not correct. I am just a custodian of the DNA passed onto me” (R12).

Additionally, respondents expressed concerns about the potential misuse of genomic data arising from patient ownership that would not occur if patients were simply able to control and decide who uses their data through existing consent processes. One clinical geneticist stated, “This is an issue of protecting patients from agents who might misuse their data. I am concerned that if individuals are given their genome data, they will hand it on to organisations that will misuse it without their [patients’] knowledge” (C5). This implies that respondents who disagreed that patient should own their genomic data may also believe that storing the data in a centrally located server is safer for patients than providing that data to patients directly.

#### Enablers and barriers in implementing patient data ownership

Four themes were identified in how respondents perceived of enablers and barriers to implementing patient data ownership in Australia. They were “patient readiness”, “genomics workforce”, “state of health system infrastructure”, and “coordinated policy environment”.

The theme of “patient readiness” refers to the state of patients’ knowledge and awareness about their own genomic data and the practice of clinical genomics, in general. Respondents perceived that patients’ current understanding of their genomic data could be a barrier to rollout mechanisms for them to responsibly and safely owning their data. Specific concerns about the utility of genomic data and its interpretation by patients were raised. A genetic counsellor and a bioinformatician feared that patients currently do not have “the ability to interact with the data in a useful way” (G15), which could lead to “a high degree of misunderstanding and misinformation unless handled carefully” (B6). As a result, respondents highlighted the need for education pathways to enable patient data ownership for both patients and professionals (as explored in the next theme). One researcher wrote, “there needs to be a pathway for educating people on safe ways to manage their genomic data” (R35). Additionally, clinical geneticists and genetic counsellors underscored the implementation of patient data ownership should be coupled with professional support.

In the “genomics workforce” theme, respondents explained that because clinical genomics is a relatively new field, there were not sufficient health professionals with genomics skills and knowledge to support the implementation of patient data ownership. As this should be enabled through appropriate professional education and support, the required genomic workforce should be expanded, particularly around “patient interface and more genetic counselling” (G40). Respondents cautioned of potential barriers in creating such a genomic workforce because this “will take several years of education to improve the general level of genetic and genomic literacy across non-genetic health professionals” (G23), which will require a long-term strategic investment.

The theme of “state of health system infrastructure” includes enablers and barriers relating to the technology and infrastructure required to support the implementation of patient data ownership. Bioinformaticians and researchers emphasised that the required technology to support patients owning their data is currently available, as one bioinformatician stated, “all of the technology and data management tools exist for this to happen” (B11). Researchers agreed that systems were in place to support this in research settings and identified the need to pilot these systems in clinical settings before rolling them out for mass uptake. However, for wider implementation of patient data ownership, respondents pointed to a key barrier of insufficient data storage and infrastructure. One genetic counsellor expressed, “we don’t have enough storage capacity to store such large data at the moment for people to ‘own’ their data” (G32). Similarly, a researcher commented that “Australia needs the right infrastructure to help people store and manage their data” (R9). Another barrier raised related to the challenges associated with embedding a user interface in the data storage system. This was expressed by a genetic counsellor, “the major barrier is online health system infrastructure is nowhere near ready to allow dynamic, interactive engagement to people’s genomic data” (G22).

In the theme of “coordinated policy environment”, respondents identified the lack of relevant legislation to support patient data ownership. One genetic counsellor commented that a barrier is “the time needed for developing appropriate protocols and legislation [and] implementing those at the federal and state levels” (G43). According to one researcher, there is “no legislation that guides data sovereignty” (R21). Most importantly, the ambiguity around patient ownership discussed earlier needs to be tackled, and as one researcher stated, “the legal and regulatory implications of data ownership as intellectual property of the individual vs. the lab generating the data needs to be resolved” (R3). The laboratory ownership model for genomic data was also discussed by respondents who expressed disagreement towards patients owning their data (under “copyright” concerns in the previous section).

## Discussion

As noted earlier, one aim of this research is to respond to the growing literature that suggests patient data ownership, enabled by blockchain technology, as the solution to balancing the safeguarding of patient privacy and data sharing. In so doing, we engaged with Australian genomic professionals to explore their views on patient data ownership and the associated enablers and barriers to implementing it in Australia. There are three key findings generated from this study.

First, the majority of respondents across all four professional groups agreed that patients have rights to data ownership, measured as their rights to “own”, “control”, and “decide who uses data”. Assessing patient data ownership across these three items was useful for eliciting some of the nuances in how ownership is interpreted by professionals working in clinical genomics. As the descriptive analysis shows, more bioinformaticians and researchers (“behind-the-scenes” professionals) agree to all three items of ownership than clinical geneticists and genetic counsellors (“patient-facing” professionals). In particular, more patient-facing professionals expressed their disagreement (29% clinical geneticists and 10% genetic counsellors) or uncertainty (6% clinical geneticists and 26% genetic counsellors) towards patients having rights to “own their data”, compared to the other two items. The hypothesis testing confirmed that “behind-the-scenes” professionals had significantly more favourable perceptions towards patient data ownership than “patient-facing” professionals.

Additionally, some patient-facing professionals, particularly clinical geneticists, expressed the view that patients owning their data may place patients at a greater direct risk of their data being misused. This finding is useful as it identifies how patient data ownership might create additional, if unintended, risks to patients that would need to be appropriately mitigated in exploring a safe and reliable patient data ownership model, along with secondary and tertiary risks. For patient-facing professionals, granting patient data ownership without adequate professional support goes against the nature of work, as one of their roles is to help patients to make decisions. A similar finding was documented in a study by Walter and Lopez [45] that examined the adoption of advanced clinical information technology by physicians to advise patients. These researchers found that physicians had unfavourable responses towards the proposed technology because they perceived the technology would limit the professional support needed to support patients to make decisions.

Second, our findings reiterate the need for clarity on the legal implications of patient data ownership. Respondents who disagreed that patients have rights to data ownership still believed that patients should be able to control their data and who uses it. Although this was only a minority of the respondents in this research, the nature of their disagreement with patient data ownership raises specific concerns about “copyrighting” of genomic data and the role of laboratories in generating the information derived from genomic data. In Australia, the Copyright Act of 1968 indicates that laboratories own patients’ genomic data as they invest the resources and skills to convert the data into usable information. This view is consistent with genomic data being co-constructed through a process involving clinicians, laboratory professionals, and other health workers [18, 20]. Among those respondents who disagreed with patient data ownership, concerns regarding collective familial ownership of genomic data were also raised. Families owning their DNA has been a contested ethical topic for a long time in the field of genetics and genomics e.g., [46, 47]. The World Health Organisation in their 1997 report proposed that DNA data should have familial ownership as a way to address ethical tension surrounding individuals owning their DNA [48]. If a responsible patient data ownership model is to be designed and implemented, the question of legal ownership and the associated rights vested in patients and other actors in the clinical genomics system does need to be clarified.

Third, both bioinformaticians and researchers identified that the technology to enable patient data ownership is already available to support piloting and upscaling. Respondents discussed the importance of creating a user-friendly and dynamic interface for patients to manage their data. As data governance has been a topic of interest globally, we argue that such an interface and the rights and responsibilities associated with accessing it should be harmonised across jurisdictions, not only across Australian states and territories, but where practicable, across countries so that the same knowledge products can be mobilised internationally for wider public benefit. The difficulty with storing data in large volumes came up as a barrier. The proponents of self-sovereign identity claim that data storage is a problem with centralised systems and that blockchain technology enabled decentralised systems can be an alternative solution [17, 49]. Our survey did not directly address this question; hence, this solution, we suggest, needs further research.

There are some limitations that warrant future research. This study was exploratory in nature, and hence targeted only four groups of professionals. The survey was sent out to potential participants and invited their response voluntarily. There may be a non-response bias in our data as it is likely that interested respondents with views about patient data ownership (whether positive or negative) may have been more motivated to participate. Given the exploratory nature of the research, even in the event of self-selection or non-response bias, the baseline findings remain useful for informing future research directions with broader and more representative samples. We further acknowledge that the perceptions of respondents may differ based on the age-groups or capacity of patients they treat, particularly of clinical geneticists and counsellors. We suggest future research collect this information.

## Conclusion

In conclusion, although the majority of respondents in our research expressed a favourable response towards patient data ownership, some patient-facing professionals identified potential risks of extending patient rights from controlling and deciding who uses their data to full patient ownership. Our study suggests implementing patient data ownership requires systemic interventions, ranging from public awareness, investment in the existing workforce, policy development, and infrastructure development. We also argue that these findings translate beyond patient contexts, such as citizens donating genetic data for research purposes. Additionally, policy development will be essential to managing the current ambiguity around how the term “ownership” is understood in relation to genomic data and to allow pathways for infrastructure development. Finally, as perceptions of professionals towards patient data ownership vary, tailor-made strategies are needed to address gaps and enable sharing multiple views and experiences among all stakeholders as part of co-developing clinical genomics systems.

### Supplementary information

Supplementary Information

## Data Availability

The data are not available for public due to ethics considerations.
