# Supplementary material for: Balancing the safeguarding of privacy and data sharing: perceptions of genomic professionals on patient genomic data ownership in Australia
Source: Eur J Hum Genet. 2023 Jan 11;32(5):506–12. doi: 10.1038/s41431-022-01273-w (PMC11061115; doi:10.1038/s41431-022-01273-w)
Supplement: Supplementary file 1 — Supplementary Information [file 41431_2022_1273_MOESM1_ESM.docx]

Supplementary information

Balancing the safeguarding of privacy and data sharing: Perceptions of genomic professionals on patient genomic data ownership in Australia

**Roles of four professionals**

In general, clinical geneticists order a genomic test, genetic counsellors organise and perform patient consultations, bioinformaticians run genome sequencing, and researchers study new variants and treatment options concerning genetic diseases. Clinical geneticists and genetic counsellors mostly work at hospitals and genetic clinics. Bioinformaticians work at laboratories and research institutions. Researchers work at research institutions.

**Packages used for the analysis**

The table below presents the R packages used in data analysis.

Supp. Table 1. Packages used for data analysis

| **Packages** | **Description** |
| --- | --- |
| *tidyverse* (1) | To clean and format the data (quantitative and qualitative) |
| *psych* (2) | To perform statistical analysis |
| *likert (3)* | To visualise like scale data |
| *RQDA (4)* | To analyse qualitative data |

**Means and standard errors**

The table below presents the mean values and standard errors across four professional groups that shows their perceptions towards patient data ownership.

Supp. Table 2. Means and standard errors

| **Professionals** | **Mean** | **Standard Error** |
| --- | --- | --- |
| Clinical geneticists | 5.53 | 0.238 |
| Genetic counsellors | 5.93 | 0.127 |
| Bioinformaticians | 6.75 | 0.137 |
| Researchers | 6.48 | 0.137 |

**ANOVA test results**

The table below shows the results of ANOVA, performed to test whether four groups of professionals have different perceptions towards patient data ownership.

Supp. Table 3. ANOVA test results

|  | **DF** | **Sum Sq** | **Mean Sq** | **F value** | **p value** |
| --- | --- | --- | --- | --- | --- |
| Professionals (clinical geneticists, genetic counsellors, bioinformaticians, researchers) | 3 | 19.16 | 6.387 | 9.41 | <.001*** |
| Residuals | 110 | 74.66 | 0.679 |  |  |

**Tukey’s test results**

The table below shows the results of Tukey’s test.

Supp. Table 4. Tukey's test results

| **Comparison** | **Difference** | **Lower** | **Upper** | **p adj** |
| --- | --- | --- | --- | --- |
| Genetic counsellors -> clinical geneticists | 0.395 | -0.209 | 1.000 | 0.33 |
| Bioinformaticians -> clinical geneticists | 1.225 | 0.507 | 1.942 | <.001 |
| Researchers -> clinical geneticists | 0.952 | 0.296 | 1.609 | 0.001 |
| Bioinformaticians -> genetic counsellors | 0.829 | 0.248 | 1.410 | 0.001 |
| Researchers -> genetic counsellors | 0.557 | 0.053 | 1.060 | 0.02 |
| Researchers -> bioinformaticians | -0.272 | -0.907 | 0.362 | 0.67 |

**References**

1. Wickham H. tidyverse: Easily Install and Load the 'Tidyverse'. R package version 1.2.1. 2017.

2. Revelle W. psych: Procedures for Personality and Psychological Research. Illinois, USA: Northwestern University; 2020.

3. Bryer J, Speerschneider K. likert: Analysis and Visualization Likert Items. R package version 1.3.5.; 2016.

4. Huang R. RQDA: R-based Qualitative Data Analysis. R package version 0.3.1. 2018.
